# Plasma tRNA-derived small RNAs signature as a predictive and prognostic biomarker in lung adenocarcinoma

**DOI:** 10.1186/s12935-022-02481-6

**Published:** 2022-02-03

**Authors:** Jun Wang, Xianyu Liu, Weifang Cui, Qun Xie, Wei Peng, Heng Zhang, Yang Gao, Chunfang Zhang, Chaojun Duan

**Affiliations:** 1 Department of Thoracic Surgery, Xiangya Hospital, Central South University, Changsha, 410008 Hunan People’s Republic of China; 2 Hunan Engineering Research Center for Pulmonary Nodules Precise Diagnosis & Treatment, Changsha, 410008 Hunan People’s Republic of China; 3 Department of Oncology, Xiangya Hospital, Central South University, Changsha, 410008 Hunan People’s Republic of China; 4 Department of Ultrasonic Imaging, Affiliated Hospital of Hunan Traditional Chinese Medicine Research Institute, Changsha, 410006 Hunan People’s Republic of China; 5 Department of Oncology, Hunan Provincial People’s Hospital, the First Affiliated Hospital of Hunan Normal University, Changsha, 410006 Hunan People’s Republic of China; 6 Institute of Medical Sciences, Xiangya Hospital, Central South University, Changsha, 410008 Hunan People’s Republic of China; 7 Xiangya Lung Cancer Center, Xiangya Hospital, Central South University, Changsha, 410008 Hunan People’s Republic of China; 8 National Clinical Research Center for Geriatric Disorders, Changsha, 410008 Hunan People’s Republic of China

**Keywords:** Lung adenocarcinoma, tsRNAs, tRFs, Network, Diagnostic biomarker

## Abstract

**Background:**

The prevalence of lung adenocarcinoma (LUAD) has increased, thus novel biomarkers for its early diagnosis is becoming more important than ever. tRNA-derived small RNA (tsRNA) is a new class of non-coding RNA which has important regulatory roles in cancer biology. This study was designed to identify novel predictive and prognostic tsRNA biomarkers.

**Methods:**

tsRNAs were identified and performed differential expression analysis from 10 plasma samples (6 LUAD and 4 normal, SRP266333) and 96 tissue samples (48 LUAD and 48 normal, SRP133217). Then a tsRNA-mRNA regulatory network was constructed to find hub tsRNAs. Functional enrichment analysis was performed to infer the potential pathways associated with tsRNAs. Afterwards, a Support Vector Machine (SVM) algorithm was used to explore the potential biomarkers for diagnosing LUAD. Lastly, the function of tRF-21-RK9P4P9L0 was explored in A549 and H1299 cell lines.

**Results:**

A significant difference of read distribution was observed between normal people and LUAD patients whether in plasma or tissue. A tsRNA-mRNA regulatory network consisting of 155 DEtsRNAs (differential expression tsRNAs) and 406 DEmRNAs (differential expression mRNAs) was established. Three tsRNAs (tRF-16-L85J3KE, tRF-21-RK9P4P9L0 and tRF-16-PSQP4PE) were identified as hub genes with degree > 100. We found Co-DEmRNAs (intersection of DEtsRNAs target mRNAs and differentially expressed mRNAs in LUAD) were engaged in a number of cancer pathways. The AUC of the three hub tsRNAs’ expression for diagnosing LUAD reached 0.92. Furthermore, the qPCR validation of the three hub tsRNAs in 37 paired normal and LUAD tissues was consistent with the RNA-Seq results. In addition, tRF-21-RK9P4P9L0 was negatively associated with LUAD prognosis. Inhibition of tRF-21-RK9P4P9L0 expression reduced the proliferation, migration and invasion ability of A549 and H1299 cell lines.

**Conclusion:**

These findings will help us further understand the molecular mechanisms of LUAD and contribute to novel diagnostic biomarkers and therapeutic target discovery.

**Supplementary Information:**

The online version contains supplementary material available at 10.1186/s12935-022-02481-6.

## Background

Lung cancer is responsible for a significant number of fatalities each year, with lung adenocarcinoma (LUAD) becoming the most common pathological type of lung cancer [1, 2]. The 5-year survival rate for LUAD is less than 20%, however early detection and therapy can significantly increase it [3, 4]. Therefore, there is a growing need to identify molecular markers for the diagnosis of LUAD, which can aid in early detection and improve LUAD prognosis.

As an important component of the biological sample pool, blood samples are rich in biomolecules that can be used for disease diagnosis, stage identification and prognosis prediction. Compared with tissue samples, blood samples have the advantages of easier access, continuous sampling and higher patient acceptance. Therefore, analyzing biomarkers found in both tissue and blood samples can improve the accuracy and convenience of cancer detection.

Transfer RNA (tRNA)-derived small RNAs (tsRNAs) are a new class of small non-coding RNAs (sncRNAs) that have recently been found as a result of advances in high-throughput sequencing and bioinformatics analysis. They are split into two types based on cleavage site and length: tRNA derived fragments (tRFs) and tRNA-derived stress-induced RNAs (tiRNAs, also known as tRNA-half) [5]. 5-tRF, 3-tRF, 1-tRF, and i-tRF are tRFs that come from mature or precursor tRNAs that are 14–30 nucleotides (nt) in length. 5′-half and 3′-half tiRNAs, 29-50nt in length, are formed by explicit cleavage of mature tRNA anticodon loop under stress conditions [6–10]. Growing evidence suggests that tsRNAs can influence the emergence and progression of numerous diseases and cancers by participating in biological processes such as gene silencing, protein translation, etc. [5, 11–15]. For example, tRF3E, which is downregulated in human epidermal growth factor receptor 2 (HER2) positive breast cancer, can interact with nucleolin to suppress p53 mRNA translation, thereby restraining the development of breast cancer [16]. In non-small cell lung cancer (NSCLC), tRF-Leu-CAG is upregulated in tissues, cell lines, and sera, which results in facilitating NSCLC progression by promoting cell proliferation [17].

Despite accumulating evidence revealing that tsRNAs can be exploited as cancer diagnostic biomarkers and therapeutic targets [18–20], the roles of tsRNA in LUAD remain largely unknown. Due to advances in sequencing and bioinformatics, we are able to use public databases to investigate new LUAD biomarkers and therapeutic targets. Here, we used raw sequencing data from the Sequence Read Archive (SRA) and data from the Cancer Genome Atlas (TCGA) to analyze differentially expressed tsRNAs in LUAD, and build a tsRNA-mRNA network to find hub tsRNAs, and used machine learning methods to create predictive models to find tsRNAs that can be used as biomarkers for diagnosis and therapy targets in LUAD.

## Materials and methods

### Workflow

The workflow is visualized in Additional file 8: Fig. S1. All data are retrieved from public databases. The LUAD miRNA-Seq samples were searched and downloaded from NCBI SRA database in April 18, 2021 (keywords: 'LUAD,' 'Human species,' and 'miRNA-Seq'). To minimize confounding factors, only plasma samples were collected; exosomes, blood cells, and other samples were not included. Patients who had just undergone surgery, as well as those who were undergoing chemotherapy or radiotherapy, were excluded from the study. For further analysis, 10 plasma samples (6 LUAD and 4 normal, SRP266333) and 96 tissue samples (48 LUAD and 48 normal, SRP133217) were included for further [21, 22]. These high-throughput sequencing data was downloaded by SRA Toolkit. Then, the raw reads were quality checked by FASTQC version 0.11.9. Next, TrimGalore version 0.6.6 was used to trim the small RNA sequencing adaptor and filter the reads using the command ‘-q 20—phred33—stringency 3—length 14'. Finally, MINTmap v2.0 was used to collapse reads, reference alignment, and quantify expression with default parameters and human GRCh37 dataset [23]. The dataset includes genome and annotation files which were obtained from different databases, such as tsRNAs that were gathered from the GtRNAdb database, miRNAs from miRbase, snoRNAs from snoRNABase, and genome, rRNA, ncRNA, and mRNA were retrieved from the Ensembl. We also used Mirge3.0 to perform read distribution analysis [24]. T-test was used to evaluate the difference in the proportion of each type of reads between normal and LUAD samples with significance level defined as P < 0.05.

### Construction of the tsRNAs-mRNA regulatory network

Differentially expressed tsRNAs (DEtsRNAs) were identified utilizing DESeq2 software (version 1.26.0), with DEtsRNAs defined as |log twofold change|> 1 and adjusted P value < 0.05. Using intersecting studies, the consistent DEtsRNAs (Co-DEtsRNAs) between plasma and tissues were determined. The tRFTar website was used to retrieve the target mRNAs of Co-DEtsRNAs [25]. By using |log twofold change|> 1 and a q-value below 0.05, the differentially expressed mRNAs (DEmRNAs) of TCGA LUAD were identified using the Gene Expression Profiling Interactive Analysis (GEPIA) website [26]. The overlapping mRNAs (Co-DEmRNAs) between target mRNAs and DEmRNAs were then identified using intersection analysis. The R package EnhancedVolcano version 3.13 was used to create volcano graphs. The UpSet plot was generated on http://www.ehbio.com/test/venn/#/. The tsRNAs-mRNA regulatory network was constructed using Co-DEtsRNAs and Co-DEmRNAs via cystoscope version 3.8.2 and Gephi 0.9.2. The hub tsRNAs were distinguished by degree > 100.

### Functional enrichment analysis and confirmation of hub genes

Metascape server was used to execute the Gene Ontology (GO), Kyoto Encyclopedia of Genes and Genomes (KEGG), Wikipathway, and Reactome Gene Sets enrichment analysis [27]. The String database was used to investigate the protein–protein intersection (PPI) network of Co-DEmRNAs. The MCODE technique was used to separate the gene cluster (highly linked areas) [28]. The core network was created using the cytoHubba tool in Cytoscape and the Maximum Clique Centrality (MCC) algorithm [29].

### Biomarker gene identification

The support vector machine (SVM) technique was used to categorize normal and LUAD samples based on the expression counts of the top three hub tsRNAs in the network. The model's performance was assessed using cross-validation. The Python scikit-learn package was used to conduct all of the model training, testing, cross-validation, and prediction.

### Patients and ethical statement

LUAD tissues and normal lung tissues (at least 5 cm from the tumor edge) were acquired from the Department of Thoracic Surgery, Xiangya Hospital of Central South University. The clinicopathological data was provided in Additional file 1: Table S1. The experiments were approved by the ethics committee of the Xiangya Hospital of Central South University.

### RT-qPCR

TRIzol reagent (Invitrogen, Carlsbad, CA) was used to extract RNA from tissues and cell lines. The TransScript One-step gDNA Removal and cDNA synthesis SuperMix kit (TransGen Biotech, Beijing, China) was used to generate first-strand cDNA. U6 was used as internal control for tsRNAs. The primers for U6, tRF-16-L85J3KE, tRF-21-RK9P4P9L0 and tRF-16-PSQP4PE were purchased from Ribo (Guangzhou, China) using the stem-loop method. GAPDH was used as internal control for Notch1 expression level detection. The primers for GAPDH and Notch1 were purchased from Tsingke (Beijing, China) using tailing method. The relative levels of RNAs were calculated using the comparative CT (2 − ΔΔCT) method. Primers used in the study were listed in Additional file 2: Table S2.

### Cell culture and RNA interference

Cell lines (Hcc827, A549, H1299, H1975, PC-9) used in the study were purchased from the Chinese Academy of Sciences Cell Bank (Shanghai, China). All cells were seeded in Roswell Park Memorial Institute (RPMI) 1640 media (Gibco, Carlsbad, USA) with 10% fetal bovine serum (BI, Israel) and 2% penicillin–streptomycin (HyClone, Logan, UT, USA) at 37 °C in the presence of 5% CO2. The cells were used within 10 passages. tRF-21-RK9P4P9L0 inhibitors was purchased from Tsingke. The inhibitors were transfected using Lipofectamine® 3000 (Invitrogen, CA, USA), following the guidelines of the manufacturer.

### Cell Counting Kit-8 and migration and invasion assays

Cell proliferation capacity was assessed using the cell counting kit-8 (Beyotime Biotechnology, Shanghai, China). 96-well plates were seeded with 3000 cells per well and recorded at 0, 24, 48, and 72 h.

Cell migration and invasion ability were assessed using transwell assays. In a 24-well plate, 40,000 cells cultured in serum-free medium were added to the upper chamber covered with (invasion) or without (migration) Matrigel (BD, USA). 1640 medium containing 10% FBS was added to the lower chambers. Twenty-four hours later, fixed with 4% paraformaldehyde and stained with 0.1% crystal violet, then 5 fields were randomly selected, numbered, and photographed under the microscope.

### Statistical analysis

GraphPad Prism 8.0 and SPSS 22.0 were used to conduct all statistical analyses. The information is presented in the form of a mean and standard error of the mean (SEM). Statistical significance was defined as a P value of less than 0.05.

## Results

### Read distribution analysis of miRNA-Seq in lung tissues and plasma from patients with LUAD

**Fig. 1:**
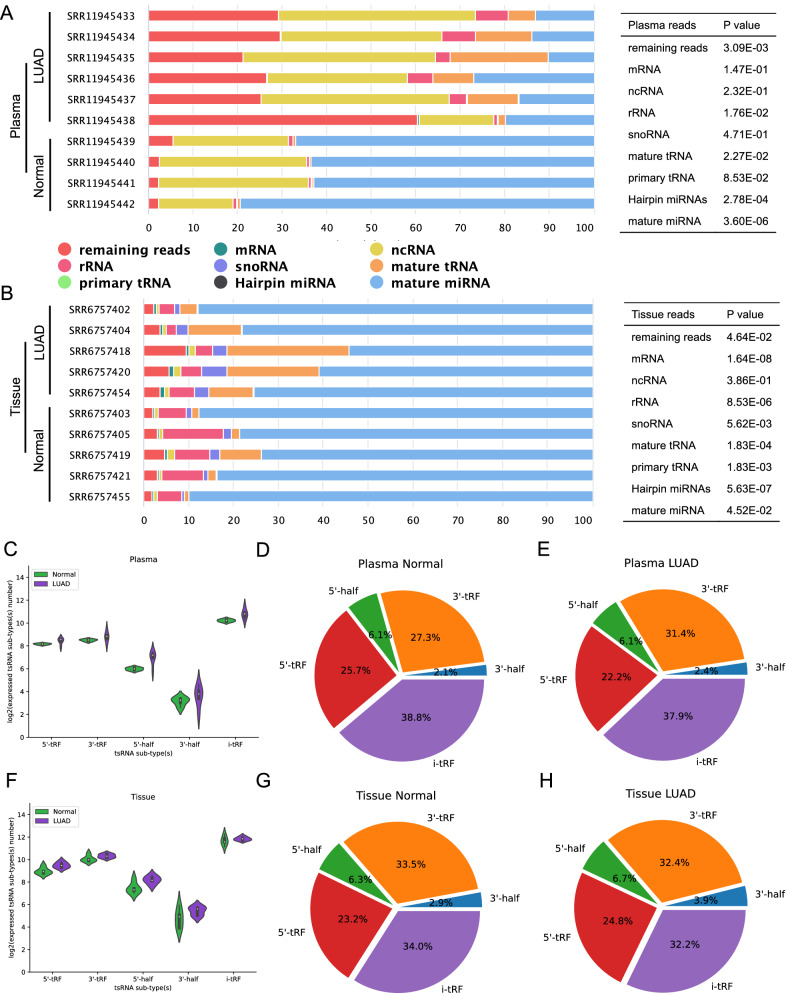
Read distribution of small RNAs and tsRNA sub-type in plasma and tissue of normal people and LUAD patients. **A** Small RNA distribution in plasma of normal people and LUAD patients, the right panel displayed the P value of the difference in the proportion of each type of reads between normal and LUAD samples; **B** Small RNA distribution in tissues of normal people and LUAD patients, the right panel displayed the P value of the difference in the proportion of each type of reads between normal and LUAD samples; **C** Expressed tsRNAs sub-type numbers in plasma of normal people and LUAD patients using violin illustration; percentage of tsRNAs sub-type numbers in plasma of normal people (**D**) and LUAD patients (**E**) using pie chart; **F** expressed tsRNAs sub-type numbers in tissues of normal people and LUAD patients using violin illustration; percentage of tsRNAs sub-type numbers in tissues of normal people (**G**) and LUAD patients (**H**) using pie chart

To investigate the tsRNA profiles in depth, we assessed at the read distribution of various types of small RNAs in plasma and tissues, including microRNA (miRNA), Ro-associated Y RNA (yRNA), tRNA, ribosomal RNA (rRNA), and non-coding RNA (ncRNA). There was a significant variation in the distribution of small RNA components between normal people and LUAD patients, as illustrated in Fig. 1A, B (10 tissue samples were randomly selected), whether in plasma or tissue. The reads proportion of all tissues samples is provided in Additional file 8: Figure S2. In contrast to tissues (whether normal or LUAD tissues), where miRNA is always a substantial component, the amount of miRNA in plasma differed significantly between normal and LUAD patients' samples. In addition, plasma LUAD samples had a higher percentage of remaining reads than normal plasma, but normal and LUAD tissues had about the same percentage of remaining reads.

Furthermore, the amount of tRNA in plasma and tissues differed between normal people and LUAD patients. As shown in Fig. 1A, B, tRNA content varied greatly: a small number of reads may be mapped to mature tRNA in normal plasma samples, whereas the proportion of tRNA in LUAD patients' plasma is substantially greater than in normal samples. As we saw with plasma, the proportion of tRNA in normal tissues was substantially lower than that in tumor tissues. Normal plasma, on the other hand, had substantially lower levels of tRNA than normal tissues.

The expressed number of various tsRNA sub-types was shown in Fig. 1C, F. In LUAD patients, both plasma and tissue tsRNA had a higher expression range when compared to normal, which was consistent with the read distribution results. The same pattern of expression range among tsRNA sub-types was found in plasma and tissue samples. i-tRF had the greatest expressed numbers, followed by 3'tRF, 5'tRF, 5'-half, and 3'-half. Figure 1D, E, G, H showed the percentages of sub-types in greater detail. However, no differences in tsRNA sub-type distribution were found between normal people and LUAD patients, whether in plasma or tissues, or between plasma and tissues.

These findings demonstrated that the distribution of small RNAs, including tRNAs, differed significantly between normal and LUAD, plasma and tissues, but not tRNA sub-type.

### Identification of differentially expressed tsRNAs in tissues and plasma from normal and LUAD patients

**Fig. 2:**
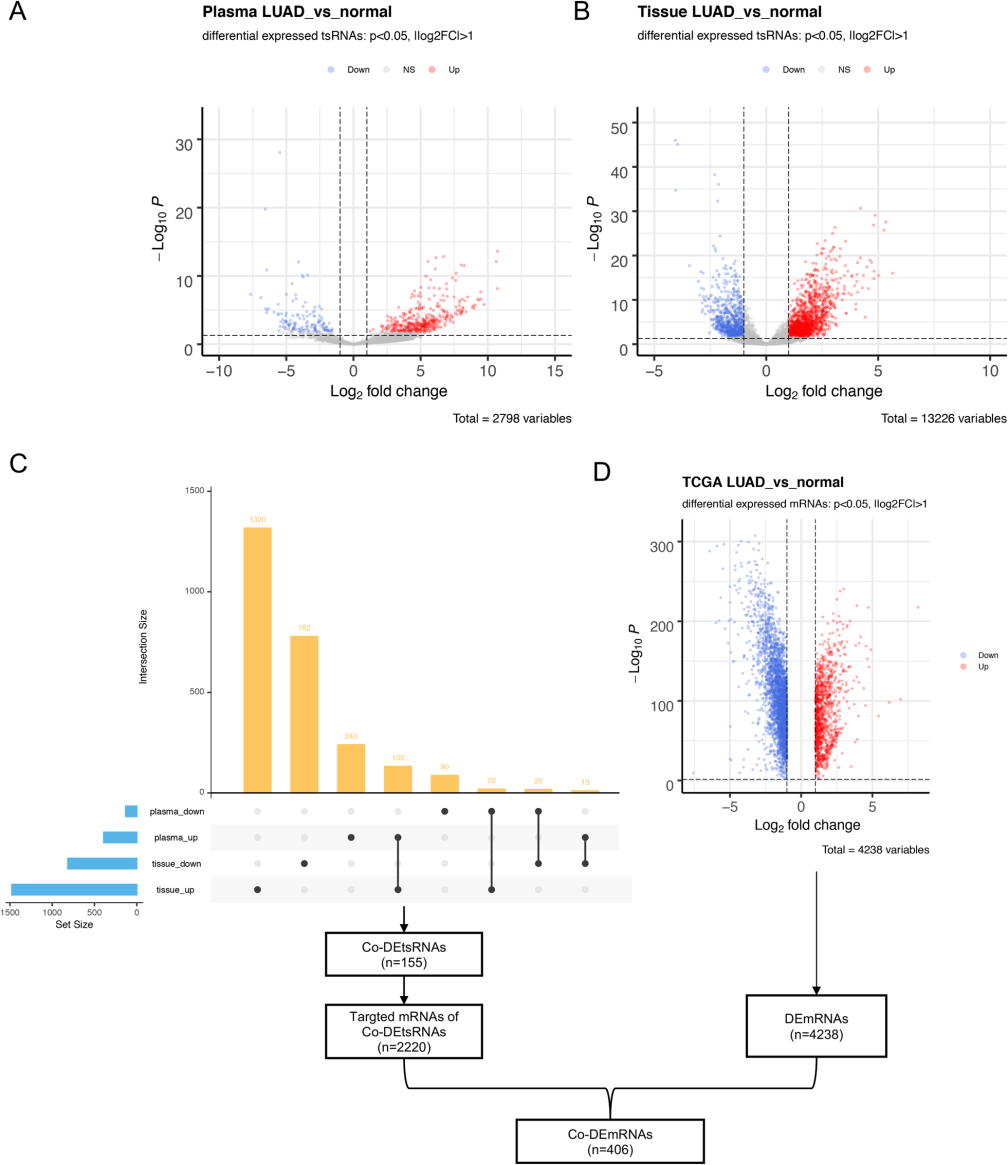
Differential expressed tsRNAs and mRNAs in LUAD. Differential expressed tsRNAs in plasma (**A**, SRP266333) and tissue (**B**, SRP133217); **C** intersection analysis of DEtsRNAs in plasma and tissues; **D** differential expressed mRNAs in LUAD using TCGA dataset. The grey dots represent genes which under significate differential expression cutoff |log2FC|> 1 and P-value < 0.05, the red plots displayed the up-regulated tsRNAs or mRNAs, the blue plots displayed the down-regulated tsRNAs or mRNAs. LUAD, lung adenocarcinoma; DE, differential expression; Co-DE, common differentially expressed

The expression levels of tsRNAs in normal plasma/tissues and LUAD plasma/tissues were determined using the pipeline we built. Only genes with an expression level larger than 10 counts in the total number of samples were maintained. Finally, 2798 and 13,226 tsRNA were found in plasma and tissue, respectively. Differentially expressed tsRNAs (DEtsRNAs) in LUAD plasma and tissues were obtained using the criteria of fold change > 2 and P value < 0.05. The differential expression analysis result had been shown using a volcano graphic (Fig. 2A in plasma, Fig. 2B in tissue). We discovered 523 DEtsRNAs in plasma, 391 of which were up-regulated and 132 of which were down-regulated. There were 2292 DEtsRNAs identified in tissues, with 1477 upregulated and 815 downregulated. These DEtsRNAs were listed in Additional file 3: Table S3.

These findings implied that LUAD patients' plasma and tissues contain a substantial number of differently expressed tsRNAs.

### Construction of the tsRNA-mRNA regulatory network

The UpSet diagram of up and down regulated tsRNA in plasma and tissue is shown in Fig. 2C. Only 155 consistent differential expression tsRNA (Co-DEtsRNAs) were detected by intersection analysis, 135 of which were up-regulated and 20 of which were down-regulated, and these were kept for further analysis. In Additional file 4: Table S4, the sequence, fold change, and sub-type of Co-DEtsRNAs in plasma and tissue were listed. To get reliable tsRNA targets mRNA, tRFTar was used in which the target genes were verified by Argonaute CLIP-Seq datasets and CLASH-Seq datasets. In total, 2220 mRNA targets were identified. We further analyzed the differentially expressed mRNAs in LUAD from TCGA and 4,238 DEmRNAs were identified (P < 0.05 and | log twofold change|> 1) (Fig. 2D). Subsequently, we determined 406 Co-DEmRNAs by intersecting the target mRNAs and DEmRNAs.

**Fig. 3:**
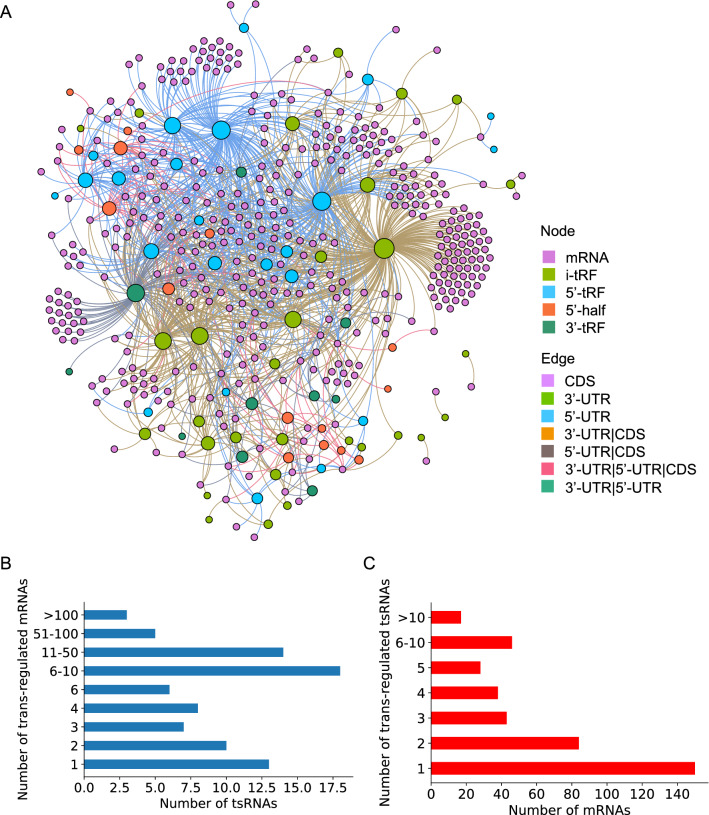
The tsRNA-mRNA regulatory network. **A** Visualized regulatory network of tsRNA-mRNA, nodes were colored to distinguish sub-types of tsRNA and mRNA, edges were colored to identify different action modes, the larger the node, the higher degree; **B** statistical analysis of the number of mRNAs which tsRNA targeted; **C** statistical analysis of the number of tsRNA targets among mRNA

We used 155 Co-DEtsRNAs and 406 Co-DEmRNAs to extract sub-network of LUAD tsRNA-mRNA network in tRFTar database. 77 tsRNAs have no target mRNA. Finally, a regulatory network comprised of 78 Co-DEtsRNAs, 406 Co-DEmRANs, and 1305 targeted connections was created. In Fig. 3A, the network is depicted. Nodes were colored differently to distinguish different forms of tsRNA and mRNA, and edges were colored differently to designate different sorts of action modes (binding sites in the 3′UTR [untranslated region], 5′UTR, or CDS [Coding DNA Sequence]). Larger node represented more neighbors. We found three hub tsRNAs in our network using the degree > 100 as a hub gene in our analysis. The most closely related tsRNA to target mRNAs was tRF-16-L85J3KE (degree = 269), followed by tsRNA tRF-21-RK9P4P9L0 (degree = 129), and tsRNA tRF-16-PSQP4PE (degree = 129). The majority of tsRNA targets were 6–10 mRNAs (Fig. 3B). Most mRNAs, on the other hand, only had one tsRNA target (Fig. 3C). tsRNAs, obviously, had more targets than mRNAs.

These results demonstrated that constructing tsRNA-mRNA regulatory networks can benefit in the discovery of essential tsRNAs in LUAD.

### Enrichment analysis of targeted mRNAs

**Fig. 4:**
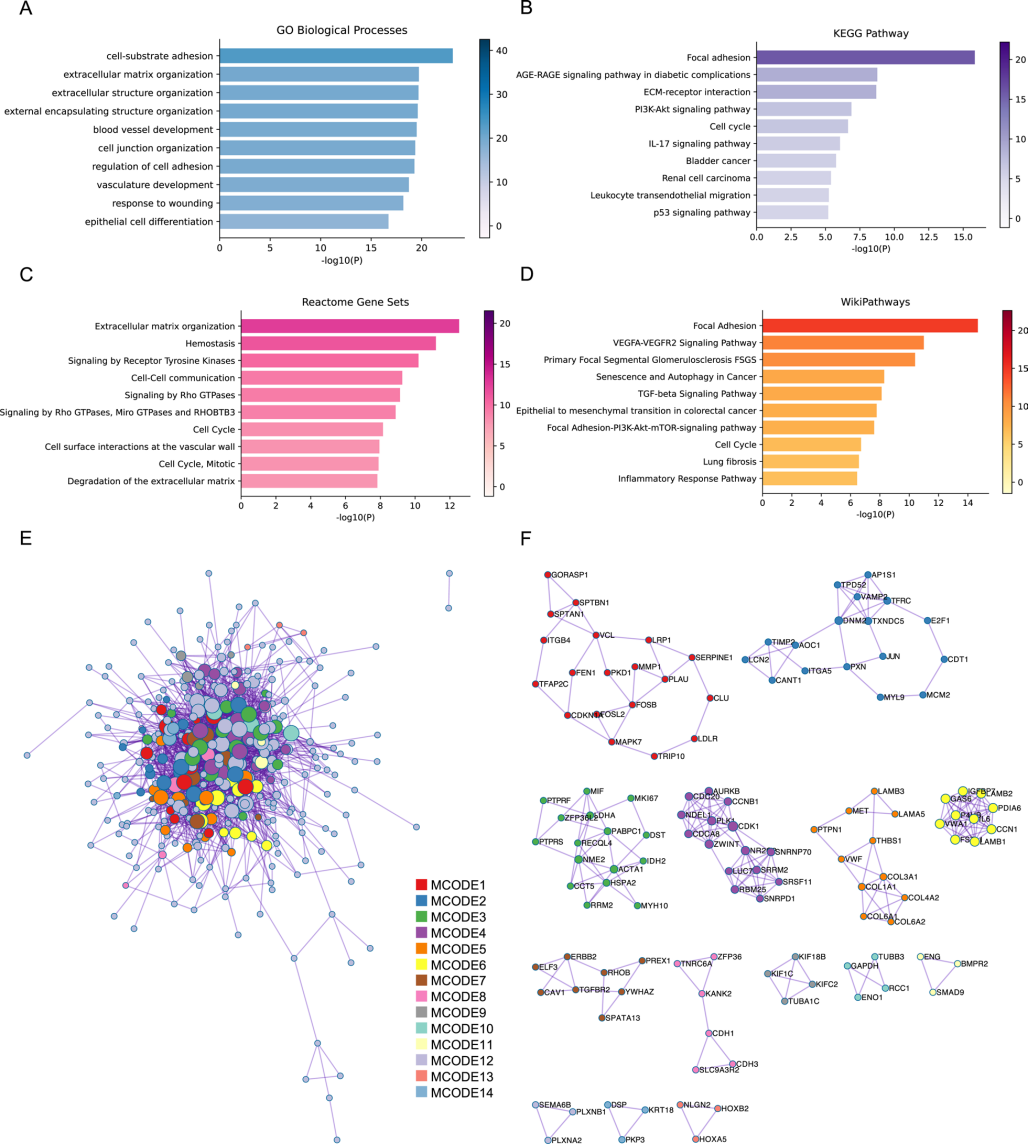
Functional enrichment of Co-DEmRNAs. **A** Graph of the top ten results from the GO analysis in terms of BP; **B** graph of the top ten enrichment pathways in KEGG; **C** graph of the top ten enrichment pathways in Recotome; **D** graph of the top ten enrichment pathways in Wikipathways; **E** PPI network of the Co-DEmRNAs, different clusters were marked with colors; **F** exhibition of 14 closely connected clusters. Co-DE, common differentially expressed; GO, Gene Ontology; BP, biological process; KEGG, Kyoto Encyclopedia of Genes and Genomes; PPI: protein–protein interaction

To investigate the probable roles of DEtsRNAs, we used GO, KEGG, Reactome, and Wikipathway enrichment analyses on these 406 Co-DEmRNAs. Figure 4A showed the top ten results from the GO biological processes. The majority of the findings were connected to the occurrence and progression of malignancies, with the majority of the findings focusing on cell adhesion. Cancer metastasis is strongly linked to cell adhesion. This might indicate a solitary metastasis that can be detected by changes in tsRNA expression. Furthermore, the KEGG pathway, Reactome, and Wikipathway enrichment analyses revealed that the cell cycle was implicated. Several cancer-related pathways, such as the p53 signaling pathway, the TGF-beta signaling pathway, and the VEGFA-VEGFR2 signaling pathway, were also included (Fig. 4B–D). Following a PPI network analysis, 349 nodes from the 406 Co-DEmRNAs were shown to be highly associated (1672 edges) (Fig. 4E). It can be classified into 14 clusters using the MCODE algorithm (Fig. 4F). Some of the clusters participated in cancer. For instance, MCODE1, which is involved in the TGF-beta signaling pathway, and MCODE3, which is involved in cell cycle and nucleotide metabolism (Additional file 5: Table S5).

These results indicated that functional enrichment analysis of tsRNA target genes can help elucidate the mechanism of tsRNAs in LUAD and identify strategies for future research.

### Biomarker identification of tsRNAs in LUAD using machine learning

**Fig. 5:**
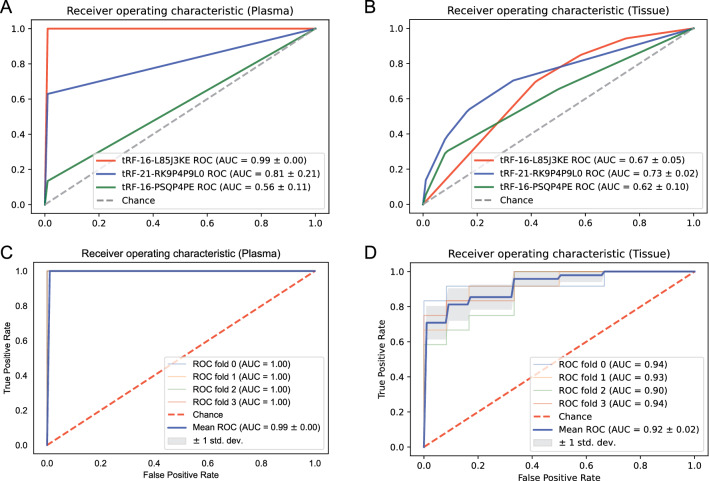
Receiver operating characteristic curves for the hub tsRNAs in plasma and tissue to distinguish LUAD patients from normal people. The AUC values obtained by using tRF-16-L85J3KE, tRF-21-RK9P4P9L0 and tRF-16-PSQP4PE individually in plasma (**A**) and tissue (**B**); The AUC values obtained in combination of tRF-16-L85J3KE, tRF-21-RK9P4P9L0 and tRF-16-PSQP4PE in plasma (**C**) and tissue (**D**). LUAD: lung adenocarcinoma; AUC: area under the receiver operating characteristic curve

We employed a fourfold cross-validation strategy to uncover candidate tsRNAs that can predict LUAD and validate our model. We developed a tsRNA expression level basis model for LUAD prediction using the SVM approach. The findings revealed that tRF-16-L85J3KE had a high likelihood of correctly classifying normal and LUAD plasma samples (Fig. 5A). Its AUC score reached 0.99, which was significantly higher than that of tRF-21-RK9P4P9L0 (0.81) and tRF-16-PSQP4PE (0.56). However, all single tsRNA in tissue can't distinguish between normal and LUAD samples well (Fig. 5B). Nonetheless, the model accuracy was significantly better when all three tsRNAs were coupled than when only one was used (Fig. 5C, D). The AUC value in the tissue increased by 0.19 to 0.92.

These findings indicated that tsRNA can be utilized as a diagnostic biomarker for LUAD, but that a single tsRNA was less efficient than a combination of multiple tsRNAs.

### Function analysis of tRF-21-RK9P4P9L0

**Fig. 6:**
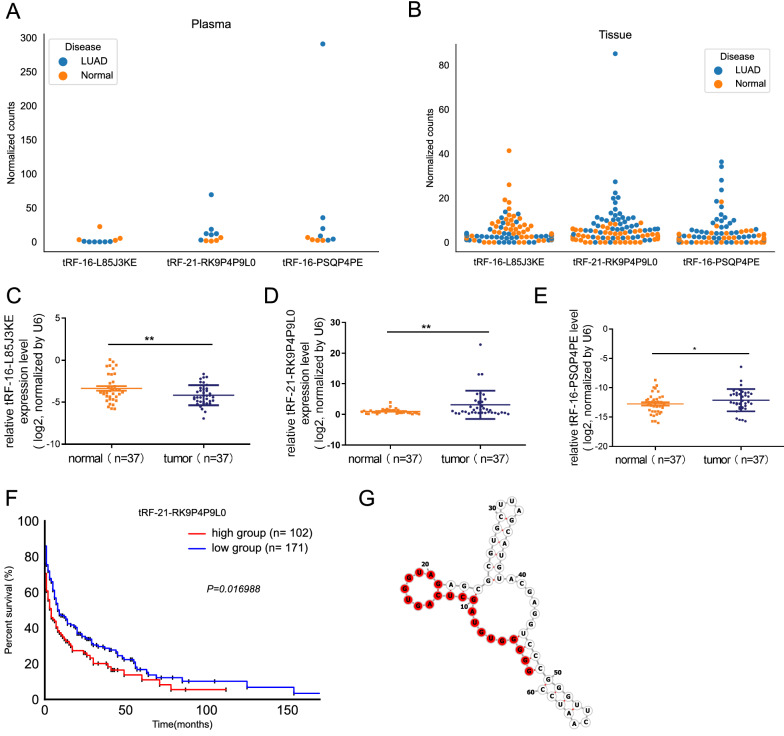
Potential tsRNAs as biomarkers. Dot plot displayed tRF-16-L85J3KE, tRF-21-RK9P4P9L0 and tRF-16-PSQP4PE expression level in plasma (**A**) and tissues (**B**); expression levels of tRF-16-L85J3KE (**C**), tRF-21-RK9P4P9L0 (**D**) and tRF-16-PSQP4PE (**E**) in LUAD tissues and paired normal tissues; **F** survival analysis of tRF-21-RK9P4P9L0 in LUAD; **G** structure and sequence of tRF-21-RK9P4P9L0

We subsequently investigated the expression characteristics of the three hub tsRNAs. i-tRF tRF-16-L85J3KE was down-regulated (log2 fold change = − 4.79, P value = 0.0063) in plasma, whereas 5′-tRF tRF-21-RK9P4P9L0 (log2 fold change = 2.94, P value = 0.0027) and 5′-tRF tRF-16-PSQP4PE (log2 fold change = 4.14, P value = 0.0075) were up-regulated (Fig. 6A). The differential expression pattern was the same in tissue, although the fold change values were smaller (Fig. 6B). Using the samples we gathered, we then validated its expression level in 37 paired LUAD tissues using RT-qPCR. As shown in Fig. 6C–E, the expression level of tRF-16-L85J3KE was decreased in tumor tissues while tRF-21-RK9P4P9L0 and tRF-16-PSQP4PE was highly elevated in tumor tissues compared to normal tissues, which was consistent with results from public data.

Only tRF-21-RKP4P9L0 was discovered to have a significant link with prognosis in LUAD (Fig. 6F, data used for analyzing the prognosis of tRF-21-RKP4P9L0 was provided in Additional file 6: Table S6), hence the properties of tRF-21-RKP4P9L0 were investigated further.

With a length of 21nt (GGGGGTGTAGCTCAGTGGTAG), tRF-21-RK9P4P9L0 was cleaved at the 5′end of tRNA. Figure 6G depicts the structure of its tRNA host.

**Fig. 7:**
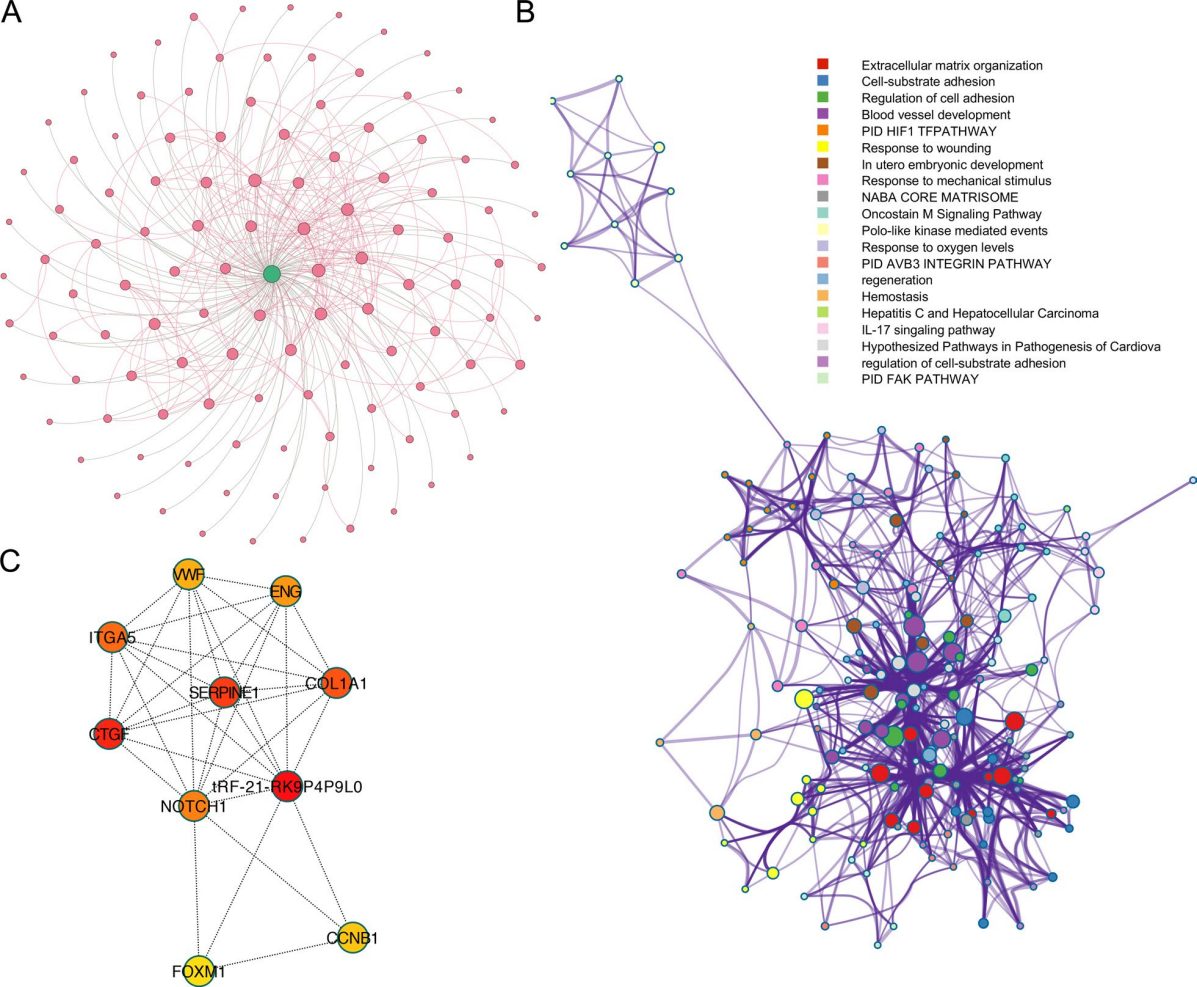
Functional analysis of tRF-21-RK9P4P9L0 using bioinformatics methods. **A** Merged network of tRF-21-RK9P4P9L0 and its target mRNAs, grey edge: tsRNA-mRNA interaction, red edge: protein–protein interaction; **B** tRF-21-RK9P4P9L0’s target gene in PPI network, individual clusters are differently colored; **C** the core sub-network of merged network

To explore the underlying mechanism of tRF-21-RK9P4P9L0, we built a target mRNA network and a PPI network to investigate its possible function. Figure 7A showed that there were 114 tsRNA-mRNA interactions and 209 PPI interactions, with the CDS region of the target mRNAs (74 CDS, 42 3′-UTR, and 5 5′-UTR) serving as the primary binding sites (Additional file 7: Table S7). We performed an enrichment analysis on the genes in the network, a distinct enriched term is presented by a different color node, Cytoscape MCC analysis was used to figure out what the core module in this network was (Fig. 7B). Figure 7C showed the extraction of a strongly linked sub-network. The network's hub was discovered to be Notch1.

**Fig. 8:**
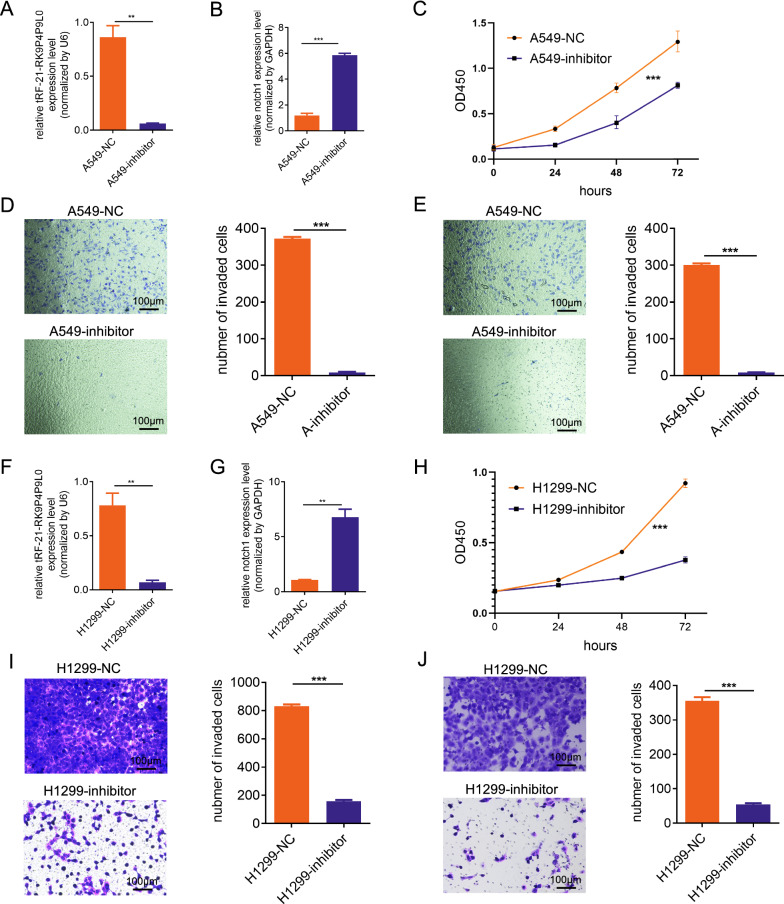
Functional analysis of tRF-21-RK9P4P9L0 in LUAD cell lines. **A** tRF-21-RK9P4P9L0 expression level in A549-NC and A549-tRF-21-RK9P4P9L0; **B** Notch1 expression level in A549-NC and A549-tRF-21-RK9P4P9L0; **C** proliferation rates of A549-NC and A549 tRF-21-RK9P4P9L0; **D** migration ability of A549-NC and A549 tRF-21-RK9P4P9L0; **E** invasion ability of A549-NC and A549 tRF-21-RK9P4P9L0; **F** tRF-21-RK9P4P9L0 expression level in H1299-NC and H1299-tRF-21-RK9P4P9L0; **G** Notch1 expression level in H1299-NC and H1299-tRF-21-RK9P4P9L0; **H** proliferation rates of H1299-NC and H1299 tRF-21-RK9P4P9L0; **I** migration ability of H1299-NC and H1299 tRF-21-RK9P4P9L0; **J** invasion ability of H1299-NC and H1299 tRF-21-RK9P4P9L0

Notch1 has been investigated to have roles in cancer cell proliferation, migration and invasion, we then explored these functional impacts of tRF-21-RK9P4P9L0 in LUAD cell lines. After detecting the tRF-21-RK9P4P9L0 expression in different LUAD cell lines, we selected A549 and H1299 for subsequent functional experiments due to their relative higher expression level (Additional file 8: Figure S3). The results demonstrated that tRF-21-RK9P4P9L0 inhibition increased Notch 1 expression level (Fig. 8A, B) and significantly reduced the proliferation (Fig. 8C), migration (Fig. 8D) and invasion (Fig. 8E) ability of A549 cells. The same impacts were observed in H1299 cell line (Fig. 8F–J).

These findings demonstrated that bioinformatics methods can be used to investigate the function of specific genes.

## Discussion

LUAD is a disease with a high mortality rate, and the molecular mechanism underlying its occurrence and progression has not been fully revealed. Previous studies of miRNA-Seq were mainly used to analyze miRNA expression profiles. Other forms of short RNAs, including tRNA, yRNA, and rRNA, may now be studied more thoroughly because of advances in bioinformatics. Several studies have disclosed tsRNAs that have been linked to the development of cancer, including lung cancer. In this study, we developed a new framework for the functional study of tsRNA in LUAD. The read distribution statistics showed that mature tRNA had a considerable proportion, and many of them were tRNA fragments. Read distribution result also showed that tsRNA content was increased in LUAD plasma and tissues compared to normal, but there were no variations in sub-type tsRNAs.

A tsRNA-mRNA regulatory network consisting of 78 Co-DEtsRNAs, 406 Co-DEmRANs, and 1305 targeted connections was established. Functional enrichment analysis of Co-DEmRNAs revealed that cancer related pathways were involved. Three hub tsRNAs (tRF-16-L85J3KE, tRF-16-PSQP4PE, and tRF-21-RK9P4P9L0) were identified by degree > 100. These three tsRNAs prediction models for LUAD were developed by a machine learning approach. The results showed that the accuracy of a single tsRNA expression used to distinguish normal from LUAD samples in both plasma and tissue was very low, so a machine learning model based on multiple expression characteristics was further built for detection. Through the model established by SVM, combining all the three hub tsRNAs can predict LUAD with an AUC 0.99 in plasma and 0.92 in tissues.

Among the three tsRNAs, only tRF-21-RKP4P9L0 was linked to the prognosis of LUAD. We built a tRF-21-RKP4P9L0 network comprising target genes, and Notch1 was at the core of the network. Considering Notch1 can contribute in cancer development [30], we validated that tRF-21-RKP4P9L0 can modify LUAD by influencing proliferation, migration and invasion.

Furthermore, we noticed that the fold change values of tsRNAs in plasma was higher than in tissue. 153 out of 155 DEtsRNAs displayed larger fold change values in plasma than in tissues. This could be due to the fact that tsRNA expression levels in plasma were substantially lower than in tissue. Plasma is easier to collect than tissue when it comes to diagnosing cancers. The most prevalent approach for determining RNA expression levels is RT-qPCR. High fold change values can improve the sensitivity of results, but lower expression levels can compromise the precision of the results. To ensure the accuracy of the diagnostic results, the critical value of tsRNAs expression level that can be identified in plasma must be determined.

However, our study has several drawbacks. To begin with, because the research on tsRNA is still in its early stages, the IDs used throughout databases are variable, as are the comparison factors, which makes tsRNA quantification more challenging. Furthermore, since the public data is derived from a variety of technological platforms, it may impact on the outcomes. Finally, future research on the mechanism of tsRNA should be performed to study them in-depth.

## Conclusions

In conclusion, we created a tsRNAs-mRNA network and discovered three hub tsRNAs. The fundamental mechanism of LUAD was investigated using functional enrichment analysis of target genes. Three tsRNAs were revealed for the diagnosis of LUAD using machine learning approach. Combining bioinformatics and experimental methods, we explored the function of tRF-21-RKP4P9L0. Our research identifies new potential diagnostic and therapeutic targets for LUAD, as well as new insights into the LUAD's pathogenesis.

## Supplementary Information

**Additional file 1: Table S1.** Clinical pathological data of 37 pairs samples used for verification of three tsRNAs’ expression.

**Additional file 2: Table S2.** Primers used in this study.

**Additional file 3: Table S3.** Differential expressed tsRNAs.

**Additional file 4: Table S4.** Consistently differentially expressed tsRNAs in plasma and tissue.

**Additional file 5: Table S5.** MCODE gene clusters.

**Additional file 6: Table S6.** Clinical pathological data used to analyze tRF-21-RKP4P9L0 prognosis

**Additional file 7: Table S7.** The tsRNA-mRNA regulated network in LUAD.

**Additional file 8: Figure S1.** Workflow of the study. **Figure S2.** Read distribution of 96 tissue samples. **Figure S3.** Expression level of tRF-21-RKP4P9L0 in LUAD cell lines.

## Data Availability

The authors confirm that the data supporting the findings of this study are available within the article and its additional materials.

## Abbreviations

LUAD: Lung adenocarcinoma
tsRNAs: TRNA-derived small RNAs
tRNA: Transfer RNA
sncRNAs: Small non-coding RNAs
tRFs: TRNA derived fragments
tiRNA: TRNA-derived stress-induced RNAs
GO: Gene Ontology
KEGG: Kyoto Encyclopedia of Genes and Genomes
PPI: Protein–protein intersection
SVM: Support vector machine
HER2: Human epidermal growth factor receptor 2
NSCLC: Non-small cell lung cancer
SRA: Sequence Read Archive
TCGA: The Cancer Genome Atlas
yRNA: Ro-associated Y RNA
rRNA: Ribosomal RNA
ncRNA: Non-coding RNA
UTR: Untranslated region
CDS: Coding DNA Sequence
