# Supplementary material for: Plasma tRNA-derived small RNAs signature as a predictive and prognostic biomarker in lung adenocarcinoma
Source: Cancer Cell Int. 2022 Feb 3;22:59. doi: 10.1186/s12935-022-02481-6 (PMC8812260; doi:10.1186/s12935-022-02481-6)
Supplement: Supplementary file 8 — Additional file 8: Figure S1. Workflow of the study. Figure S2. Read distribution of 96 tissue samples. Figure S3. Expression level of tRF-21-RKP4P9L0 in LUAD cell lines. [file 12935_2022_2481_MOESM8_ESM.docx]

**Figure legends**

**Figure S1. Workflow of the study.**

A: identify differential expression tsRNA from smRNA-Seq data. B: LUAD biomarker discovery using network and machine learning method. LUAD, lung adenocarcinoma; DE, differential expression; Co-DE, common differentially expressed; Co-mRNAs, common mRNAs.

**Figure S2. Read distribution of 96 tissue samples.**

Read distribution of 96 tissue samples (48 normal and 48 LUAD).

**Figure S3. Relative expression level of tRF-21-RKP4P9L0 in LUAD cell lines.**

Relative expression level of tRF-21-RKP4P9L0 in Hcc827, A549, H1299, H1975 and PC-9 cell lines.
